# Genome Sequence of *Staphylococcus massiliensis* Strain S46, Isolated from the Surface of Healthy Human Skin

**DOI:** 10.1128/genomeA.00553-13

**Published:** 2013-08-08

**Authors:** Rajpal Srivastav, Ajit Singh, Pramod Kumar Jangir, Chanchal Kumari, Suchismita Muduli, Rakesh Sharma

**Affiliations:** Institute of Genomics and Integrative Biology, Council of Scientific and Industrial Research (CSIR), Delhi, Indiaa; Department of Biotechnology, University of Pune, Pune, Indiab; National Chemical Laboratory, Council of Scientific and Industrial Research (CSIR), Pune, Indiac

## Abstract

*Staphylococcus massiliensis* strain S46 was isolated from the surface of healthy human skin. Here, we report the draft genome sequence of *S. massiliensis* S46 (2,447,110 bp, with a G+C content of 36.3%).

## GENOME ANNOUNCEMENT

*Staphylococcus massiliensis* strain S46 was isolated from the surface of healthy human skin and was identified as *S. massiliensis* based on the *rrs* (16S rRNA) gene sequence identity of 100% with type strain *S. massiliensis* 5402776 ^T^. *S. massiliensis* is a coagulase-negative, nonmotile, non-spore-forming bacterium that was first isolated from a human brain abscess (1) and later was proposed as a component of normal human skin microflora (2). The genome sequence of strain 5402776 ^T^ was reported recently (3). Here, we report the draft genome sequence of *S. massiliensis* S46.

The genomic DNA of *S. massiliensis* S46 was extracted from culture grown on Luria-Bertani (LB) broth. The genome of *S. massiliensis* S46 was sequenced by a whole-genome shotgun sequencing strategy using 454 GS FLX, which produced a total of 157,855 reads. The reads were assembled into 64 large (>500 bp) contigs by Newbler (version 2.6). The contigs were annotated using the prokaryotic genome automatic annotation pipeline at NCBI. The automated pipeline uses GeneMark and Glimmer (4, 5, 6) for gene prediction and compares the translated proteins with the nonredundant protein database at GenBank, Entrez Protein Clusters (7), the Conserved Domain Database (8), and COGs (9) for annotation. The genome sequence was also submitted to the RAST (10) server for annotation and identification of metabolic pathways.

The *S. massiliensis* S46 unclosed draft genome is 2,447,110 bp in length, with a G+C content of 36.3%. The genome contains 2,324 coding sequences, 56 tRNA genes, and 6 rRNA genes. The strain S46 genome has predicted genes for glycolysis, the tricarboxylic acid (TCA) cycle, fatty acid metabolism, phosphorus metabolism, iron acquisition, and organic sulfur assimilation.

A comparison with the genome sequences available at RAST showed that *Staphylococcus epidermidis* strain RP62A (score, 523) was the closest neighbor of *S. massiliensis* S46. The genes for *N*-linked glycosylation, sialic acid metabolism, thiamin biosynthesis, and trehalose uptake and utilization were present in *S. massiliensis* S46 but not in *S. epidermidis* RP62A. However, the genes for biofilm formation, quorum sensing, late competence, arginine biosynthesis, inorganic sulfur assimilation, and urea decomposition were absent in *S. massiliensis* S46 but were present in *S. epidermidis* RP62A. A detailed comparative genome analysis between the strains of *S. massiliensis* and *S. epidermidis* will reveal their metabolic differences and strategies to survive as commensals on the human skin surface.

### Nucleotide sequence accession numbers.

This whole-genome shotgun project has been deposited at DDBJ/EMBL/GenBank under the accession no. AMSQ00000000. The version described in this paper is the first version, AMSQ01000000. The 64 contigs have been deposited under the accession no. AMSQ01000001 to AMSQ01000064.

## References

[1] Al Masalma M Raoult D Roux V 2010 Staphylococcus massiliensis sp. nov., isolated from a human brain abscess. Int. J. Syst. Evol. Microbiol. 60:1066–1072 19666814 10.1099/ijs.0.006486-0

[2] Zong Z 2012 The newly-recognized species *Staphylococcus massiliensis* is likely to be part of the human skin microflora. Antonie Van Leeuwenhoek 101:449–451 21877124 10.1007/s10482-011-9635-5

[3] Roux V Robert C Gimenez G Raoult D 2012 Draft genome sequence of *Staphylococcus massiliensis* strain 5402776^T^. J. Bacteriol. 194:6984–6985 23209235 10.1128/JB.01909-12 PMC3510567

[4] Borodovsky M McIninch J 1993 GeneMark: parallel gene recognition for both DNA strands. Comput. Chem. 17:123–133

[5] Lukashin AV Borodovsky M 1998 GeneMark.hmm: new solutions for gene finding. Nucleic Acids Res. 26:1107–1115 9461475 10.1093/nar/26.4.1107 PMC147337

[6] Delcher AL Harmon D Kasif S White O Salzberg SL 1999 Improved microbial gene identification with Glimmer. Nucleic Acids Res. 27:4636–4641 10556321 10.1093/nar/27.23.4636 PMC148753

[7] Klimke W Agarwala R Badretdin A Chetvernin S Ciufo S Fedorov B Kiryutin B O’Neill K Resch W Resenchuk S Schafer S Tolstoy I Tatusova T 2009 The National Center for Biotechnology Information’s Protein Clusters Database. Nucleic Acids Res. 37:D216–D223. 10.1093/nar/gkn734 18940865 PMC2686591

[8] Marchler-Bauer A Lu S Anderson JB Chitsaz F Derbyshire MK DeWeese-Scott C Fong JH Geer LY Geer RC Gonzales NR Gwadz M Hurwitz DI Jackson JD Ke Z Lanczycki CJ Lu F Marchler GH Mullokandov M Omelchenko MV Robertson CL Song JS Thanki N Yamashita RA Zhang D Zhang N Zheng C Bryant SH 2011 CDD: a Conserved Domain Database for the functional annotation of proteins. Nucleic Acids Res. 39:D225–D229. 10.1093/nar/gkq1189 21109532 PMC3013737

[9] Tatusov RL Galperin MY Natale DA Koonin EV 2000 The COG database: a tool for genome-scale analysis of protein functions and evolution. Nucleic Acids Res. 28:33–36 10592175 10.1093/nar/28.1.33 PMC102395

[10] Aziz RK Bartels D Best AA DeJongh M Disz T Edwards RA Formsma K Gerdes S Glass EM Kubal M Meyer F Olsen GJ Olson R Osterman AL Overbeek RA McNeil LK Paarmann D Paczian T Parrello B Pusch GD Reich C Stevens R Vassieva O Vonstein V Wilke A Zagnitko O 2008 The RAST server: rapid annotations using subsystems technology. BMC Genomics 9:75. 10.1186/1471-2164-9-75 18261238 PMC2265698

